# Diageo's 'Stop Out of Control Drinking' Campaign in Ireland: An Analysis

**DOI:** 10.1371/journal.pone.0160379

**Published:** 2016-09-16

**Authors:** Mark Petticrew, Niamh Fitzgerald, Mary Alison Durand, Cécile Knai, Martin Davoren, Ivan Perry

**Affiliations:** 1 Faculty of Public Health and Policy, London School of Hygiene and Tropical Medicine, 15-17 Tavistock Place, London, United Kingdom, WC1H 9SH; 2 Institute for Social Marketing, UK Centre for Tobacco and Alcohol Studies, University of Stirling, Stirling, United Kingdom, FK9 4LA; 3 Department of Epidemiology & Public Health, Western Gateway Building, University College Cork, Cork, Ireland; Pennsylvania State University College of Medicine, UNITED STATES

## Abstract

**Background:**

It has been argued that the alcohol industry uses corporate social responsibility activities to influence policy and undermine public health, and that every opportunity should be taken to scrutinise such activities. This study analyses a controversial Diageo-funded ‘responsible drinking’ campaign (“Stop out of Control Drinking”, or SOOCD) in Ireland. The study aims to identify how the campaign and its advisory board members frame and define (i) alcohol-related harms, and their causes, and (ii) possible solutions.

**Methods:**

Documentary analysis of SOOCD campaign material. This includes newspaper articles (n = 9), media interviews (n = 11), Facebook posts (n = 92), and Tweets (n = 340) produced by the campaign and by board members. All material was coded inductively, and a thematic analysis undertaken, with codes aggregated into sub-themes.

**Results:**

The SOOCD campaign utilises vague or self-defined concepts of ‘out of control’ and ‘moderate’ drinking, tending to present alcohol problems as behavioural rather than health issues. These are also unquantified with respect to actual drinking levels. It emphasises alcohol-related antisocial behaviour among young people, particularly young women. In discussing solutions to alcohol-related problems, it focuses on public opinion rather than on scientific evidence, and on educational approaches and information provision, misrepresenting these as effective. “Moderate drinking” is presented as a behavioural issue (“negative drinking behaviours”), rather than as a health issue.

**Conclusions:**

The ‘Stop Out of Control Drinking’ campaign frames alcohol problems and solutions in ways unfavourable to public health, and closely reflects other Diageo Corporate Social Responsibility (CSR) activity, as well as alcohol and tobacco industry strategies more generally. This framing, and in particular the framing of alcohol harms as a behavioural issue, with the implication that consumption should be guided only by self-defined limits, may not have been recognised by all board members. It suggests a need for awareness-raising efforts among the public, third sector and policymakers about alcohol industry strategies.

## Introduction

The global alcohol industry, a diverse [1] yet increasingly consolidated [2] grouping of producers, distributers and related industries, has recently intensified its corporate social responsibility (CSR) activities, in ways which are ‘*instrumental to the industry’s economic interests’* [3]. Four types of CSR activities have been described: (i) sponsorship of scientific research; (ii) efforts to influence public perceptions of research findings; (iii) dissemination of scientific information, for example in conferences, books and websites, which ostensibly promote the dissemination of scientific information, but are used to support industry-favorable policy initiatives; and (iv) industry-funded public policy initiatives [3]. Other industry activities may also seek to influence policy more directly; for example industry actors have also been implicated in drafting the text of policy documents in four countries in sub-Saharan Africa [4], and in the misrepresentation of evidence to policy-makers[5].

### Alcohol industry strategies and tactics

A variety of industry-funded Social Aspects/Public Relations Organisations (SAPROs) are used to deliver on CSR activities. The establishment of the International Center for Alcohol Policies (reformed as the International Alliance for Responsible Drinking, or IARD) [6], for example describes itself as ‘a resource for all those interested in alcohol policy worldwide’, and was referred to in documents from Miller Brewing (then controlled by the tobacco company Philip Morris) as the ‘latest’ initiative that would assist ‘our sales and marketing group in an increasingly competitive marketplace’[6]. Some SAPROs such as Drinkaware [7] and Drinkwise [8] [9] focus on providing advice to the public, typically with the stated aim of helping individuals make informed decisions about their drinking; they claim to be independent, and attract government support.[7 10] Previous studies have suggested that SAPROs are part of a wider industry strategy to frame issues around alcohol harms, to promote ineffective interventions [11], and to influence the policy process, while undermining public health perspectives [5 11–14] [3] [6] [7] [8] [9] [15].

The most recent systematic review [14] found that five tactics are commonly used by the alcohol industry to influence regulation. These include ‘information’ tactics (providing or misrepresenting evidence); ‘constituency building’ (forming alliances with other sectors, organisations or the public); ‘policy substitution, development and implementation’ (e.g., Developing/promoting non-regulatory Initiatives, which are generally ineffective/less effective, in particular informational and educational programmes); ‘legal’ tactics (such as using or threatening legal action); and ‘financial incentive or disincentives’ (e.g., threatening financial withdrawal). The same review also reported that industry arguments can be grouped into five main frames–that is, ways in which industry frames issues relating to alcohol and alcohol harms. These frames are ‘regulatory redundancy’, which includes an emphasis on individual consumer responsibility, rather than regulation; a ‘legal’ frame (e.g., arguing that regulation infringes the legal rights of company); ‘negative unintended consequences’ (e.g., arguing that Regulation will result in financial or job losses); a ‘complex policy area’ frame, which industry uses to suggest that the problem would benefit from collaboration with industry, and which denigrates public health actors; and an ‘insufficient evidence’ frame, which frames the issue as one in which the scientific evidence does not support the particular intervention or policy [14]. The review concluded that every opportunity should be taken to scrutinise industry CSR activities in order to understand how and when these frames and tactics are employed.

### Diageo’s ‘responsible drinking’ campaign in Ireland: “Stop Out of Control Drinking”

One such activity is a Diageo-funded ‘responsible drinking’ campaign called “Stop out of Control Drinking” (SOOCD) which was launched in Ireland in February 2015, with the stated aim of “*changing Ireland’s culture of drinking for the better*” and making “out-of-control drinking” socially unacceptable by 2021 [16]. Levels of alcohol consumption and binge drinking (defined by Ireland’s Health Service Executive as six or more standard drinks in one session, which is the equivalent of three or more pints of beer, or six or more pub measures of spirits [17]) are among the EU’s highest, and more than half of 18–75-year-old drinkers can be classified as harmful drinkers using the World Health Organization’s AUDIT-C screening tool[18]. The SOOCD campaign was initiated with a series of advertisements, social media activity and public meetings [19], and a resultant “action plan” was published in September 2015.

The campaign has been controversial, with critics suggesting that it aims to undermine Ireland’s latest alcohol Bill, published on 3^rd^ February 2015[20]. The measures proposed in the Bill include Minimum Unit Pricing (MUP), and regulation of alcohol advertising and sponsorship (including sports sponsorship)[21]. The Bill itself is backed by a Regulatory Impact Assessment, setting out the estimated costs, benefits and impacts of the measures it proposes [22]. It also draws on an earlier (2012) report, the *Steering Group Report on a National Substance Misuse Strategy* (NSMS), which reviewed the evidence in order to identify effective policies and actions to tackle alcohol harms in Ireland[23]. The Regulatory Impact Assessment concluded that “…*the evidence base indicates that the impact of alcohol-education programmes on harmful use of alcohol is small…Evidence indicates that when these campaigns are accompanied by the imposition of higher prices/taxes or disincentives they have a direct effect in changing behaviour*” [22]. Permitting self-regulatory measures by the industry were discounted in the Regulatory Impact Assessment on the basis that the evidence shows that such approaches are ineffective. The package of measures to be implemented through the Public Health (Alcohol) Bill therefore includes supply-side measures including minimum unit pricing, the regulation of advertising and marketing of alcohol, and the regulation of sports sponsorship. These measures are consistent with the international evidence on the most effective population-based measures to reduce alcohol harms [24] [25] [26].

There has been particular concern about the involvement of Diageo on the SOOCD campaign board, and about the relationship of a number of other board members to the company. Diageo itself was initially represented on the board by its then-Director in Ireland, David Smith. Other board members also have, or have had links to, or funding from, Diageo (Table 1). Furthermore, the campaign itself is supported by public relations firm Goddard Global[27], which has had both Diageo and tobacco companies as clients, and is linked to the Common Sense Alliance, a tobacco industry lobby group [28].

**Table 1:** Board members, Diageo-funded “Stop Out of Control Drinking” (SOOCD) campaign.

Board member and other key supporters	Role, background, previous connection with Diageo/Guinness, if any; other comments. Note: It is not possible to fully ascertain board members’ past/current business/consultancy or other relationships with Diageo or the alcohol industry, as the SOOCD website includes no information on conflicts of interest.	Main data located which contributed to the analysis (i.e., interviews with SOOCD board members, and/or newspaper or other articles or blogs written by them)
Fergus Finlay	Chair of SOOCD board; CEO, Barnardos Ireland (a children’s charity). Mentor to the Arthur Guinness social entrepreneur fund	3 interviews, 2 newspaper articles written
David Smith	David Smith (Diageo Ireland, Country Director), *Resigned from SOOCD board March 25*^*th*^ *2015*	3 interviews, 1 newspaper article
Gavin Duffy	Entrepreneur & TV Presenter; has worked as an alcohol industry consultant, and for Guinness.	2 interviews, 1 newspaper article
Joanna Fortune	Clinical Psychotherapist	1 interview, 1 newspaper article
Maureen Gaffney	Author, psychologist, attached to Geary Institute, University College Dublin	Statements in news article,contribution to SOOCD workshop
Paul Gilligan	CEO of St Patrick’s Mental Health Services; *Resigned from SOOCD board*, *March 25*^*th*^ *2015*	No interviews or other data located
Dr Ciara Kelly	General Practitioner & Health Commentator, *Resigned from SOOCD board*, *March 5*^*th*^ *2015*	1 interview, 1 newspaper article
Aine Lynch	CEO, National Parents Council	Supporting statement on SOOCD website, and at SOOCD launch
Kieran Mulvey	Chief Executive of the Labour Relations Commission	No interviews or other data located
Gemma Doorly	Actress, Playwright	1 newspaper article
Simon Keogh	IRUPA (Irish Rugby Union Players’ Association). IRUPA is sponsored by Diageo.	No interviews or other data located
Krystian Fikert	Psychologist, Founder of MyMind. Former recipient of Arthur Guinness Fund award. *Resigned from SOOCD board*, *March 16*^*th*^ *2015*	No interviews or other data located
Anne Connolly	Director, Irish Smart Ageing Exchange	Statements on SOOCD website
Rob Hartnett	CEO of Sport for Business, which has Guinness as a client	1 newspaper article
Professor Briain MacCraith	President, Dublin City University, a supporting partner of SOOCD, which partners with Diageo in other activities. Wrote the SOOCD Action Plan.	No interviews or other data located
Professor Kevin Rafter	Dublin City University. Also involved in writing the SOOCD Action plan. DCU received Diageo funding, is a supporting partner of SOOCD (see above).	No interviews or other data located
Charlie O’ Connor	Former TD (member of the Irish Parliament), SOOCD board member	2 interviews

This study analyses the SOOCD campaign, and its goals and activities as publicly represented by campaign documents and social media outputs, and through board members’ statements, media interviews and newspaper articles. Its main aims were to establish to what extent criticisms of the campaign were justified, particularly criticisms about its lack of independence from Diageo; and to assess how the SOOCD campaign fits with existing evidence on alcohol industry CSR activities, particularly those which appear to be intended to influence policymaking.

## Methods

We conducted a documentary analysis of the material produced by the SOOCD campaign, using a similar approach to previous analyses of tobacco industry documents. We first identified all available material produced by the campaign and its advisory board members. This material included statements and media interviews given by board members, newspaper articles written by them, and promotional videos produced by the campaign. We also monitored the SOOCD website and SOOCD and board members’ Twitter accounts to identify other relevant data. We identified newspaper articles written by board members through a Nexis search of all Irish publications for the year preceding 16^th^ June 2015, using the term ‘stop out of control drinking’. We then used snowballing techniques and cross-referencing to identify additional material. We included in our analysis all material dating from the launch of the campaign on 12^th^ February 2015, until 30^th^ April 2015 (when most SOOCD media activity ceased). The material we identified (i.e. the data included in the documentary analysis) therefore consisted of:

1. Audio or video of media interviews with SOOCD board members (e.g. radio, YouTube or on websites) (n = 11);
2. Newspaper articles and other material written by board members and SOOCD supporters (n = 9);
3. Shorter statements and direct quotes from board members made in newspaper articles (n = 22);
4. All text and documents from the SOOCD website, including the statement of the campaign aims, the memorandum of understanding, and materials from SOOCD workshops;
5. Posts on the SOOCD Facebook timeline (n = 92) (S1 Table); and
6. Tweets posted about SOOCD from the Twitter accounts of board members (n = 340) (S1 Table).

The Facebook posts dated from 12th February 2015 to 28th April 2015; the Twitter posts dated from 12th February 2015 to 17th April 2015.

The analytic approach used standard methods widely used in the qualitative analysis of industry documents, including the use of systematic conceptual coding, and constant comparison methods with analysis of deviant cases [29] [30] [31]. To do this, all the documents (approximately 45,000 words in total) were transcribed verbatim, then open-coded independently by two researchers (MP, and one other researcher, either NF, MC, M-A D, or CK). From this, an initial set of 102 codes were agreed. These codes were applied to all the above data, and deviant cases noted. A thematic analysis was then undertaken with the codes then grouped by consensus into two broad themes and seven sub-themes. No ethical approval was required as the study involved secondary analysis of publically available qualitative data.

## Results

The findings are presented below within two broad themes: 1. “Framing the problem” (describing how board members and the campaign materials define ‘out-of-control drinking’, who it affects, and its perceived causes) and 2. “Framing the solution”, which describes the solutions recommended by board members and campaign supporters; as well as issues around the campaign’s independence from Diageo.

### 1. Framing the problem

#### (i) How board members and SOOCD campaign supporters define ‘out-of-control drinking’

The campaign focuses on ‘out of control drinking’. What this means is not clearly defined, but in one example a health-related definition is explicitly rejected by Diageo board member David Smith:

Interviewer: *Break it down to pints or cans*. *What’s ‘out of control drinking’ in your mind*?

David Smith: *Yeah*, *um*. *[pause] The way*, *ah*, *some people talk about binge drinking is 3 pints*, *which I think is potentially unhelpful here*, *that’s ah*, *one definition*. *For me…*

Interviewer: *The World Cancer Research Fund says three alcoholic drinks a day could cause liver cancer—that’s their definition*.

David Smith: *For me*, *it’s different for different people and that’s why actually the term “out of control drinking” is when your drinking is either affecting you or affecting other people* [32].

This emphasis on self-definition also appears in social media appeals, for example “*Tell us what out-of-control drinking means to you and how to put a stop to it”* (Facebook post, 16^th^ March 2015).

‘Out of control’ drinking is also presented by the SOOCD campaign as a behavioural issue: the major focus of the campaign is on the behavioural consequences of drinking, and not the health consequences, such as in these examples:

“*What we mean by stop out of control drinking*, *is… you were planning on taking your son or daughter to football training or to the swimming pool or whatever and you were too hung over*..”

(Board member Gavin Duffy [33]).

“No matter what study you read, the relationship between alcohol abuse and family breakdown, or domestic violence or gang behaviour, or almost any of the social issues that trouble us all the time, is profound”

(SOOCD board Chair, Fergus Finlay, [34]).

A rare reference to one aspect of health (though still with a behavioural focus) is made by board member and General Practitioner (GP), Dr. Ciara Kelly (board member until her resignation in March 2015):

“*Alcohol is the single biggest risk factor for death among young Irish men under 30 through assaults and accidents as well as suicides*. *Think of all those young men's families—destroyed by drink”* [35].

Overall alcohol harms are frequently framed as “negative drinking behaviours”, as in the SOOCD “Call for evidence”: *“How can schools/university-based education programmes that are offered to young people and to adults prevent negative drinking behaviours*?*”* [36].

#### (ii) Defining and redefining ‘moderate drinking’

“Moderate” drinking is frequently espoused by board members. However there is reluctance to define this, other than in terms of behaviour, as in this exchange [37] and elsewhere [36].

David Smith (Diageo board member until his resignation in February 2015):*…We would define it [moderate drinking] as ‘beware of out of control drinking’*, *we don’t really …*

Interviewer: *So anything other than out-of-control drinking is moderate*, *so if you’re not …*.*falling around*, *you’re moderate*?

David Smith: *Well*, *out-of-control means different things to…to…to different people*, *clearly…*

Interviewer: *Well*, *what’s it mean to the drinks industry*?

David Smith: *It’s all about the individual*, *at the time*, *so if you drink so much that you can’t get up in the morning and take your child to the sport that they wanted*, *even though you weren’t really drunk the night before*, *that’s too much…*.*I would say*, *ah*, *that people*, *should*, *em*, *know…how much they can drink*, *they should…ah…not step beyond that*, *and*, *and*, *really you know*, *part of the issue that we’ve got in Ireland… It’s much more about the attitude and the behaviour rather than any specific definition*.

Board member Gavin Duffy also defines drinking moderately in terms of behaviour: “*Moderate drinking involves not letting others down*” [33]. Some board members also state explicitly that moderate drinking is normal, and identify *not* drinking as abnormal [38–40]. Several also state that critics of the campaign are non-drinkers and prohibitionists. In one example SOOCD board member Gavin Duffy identified himself as a non-drinker, and then emphasised non-drinkers’ lack of entitlement to comment:

“I am not sure non-drinkers should tell others how to moderate their drinking… What sets us apart from the other campaigners is that we are not prohibitionists- we are moderationists. I have no plan to ban alcohol…”

(Gavin Duffy [41])

David Smith also contrasted the campaign’s aims of moderate consumption with what he described as “prohibitionism”, while at the same time conflating moderation with “control”, rather defining it in relation to the amount of alcohol consumed:

“*Moderate consumption of alcohol is perfectly compatible with a healthy lifestyle*, *many people drink alcohol*, *they drink so moderately*, *they’re in control*, *and there’s no issues at all*. *So they’re very*, *very different propositions there*, *and whilst there are a small number*, *of*, *I think*, *prohibitionists that are out there*, *I think the majority of people as seen by the campaign*, *have got a pretty balanced view here*.” [39]

Sport for Business, a SOOCD partner, similarly criticises unspecified groups (presumably alcohol charities and/or public health critics) claiming that they advocate prohibition:

“*Other groups employ full time executives to highlight issues around alcohol*, *but in the past month have spent more time being offended by our involvement as a group in ‘their’ debate…*. *They argue for prohibition as the only effective solution*. *Perhaps they are unaware of the abject failure that proved in the 1920’s*”. [38]

#### (iii) Who is affected: targeting youth drinking

Alcohol industry campaigns usually focus on alcohol misuse, particularly in young people, rather than on whole population approaches [11]. It is assumed that this targeted approach is favoured by industry because focusing on the extremes of age/gender/behaviour frames the problem away from population-level risk (that is, it frames it away from the larger population who consume the most alcohol in aggregate).[42] Here, too, SOOCD board members often place emphasis on children, young people and students, and young girls. (Some of the campaign adverts also focused on drunk young women, and were criticised as ‘woman shaming’ [43]).

“In a generation or two, we have gone from a drink culture to a drink society…Are you concerned that 14-year-old girls are getting vodka, pouring it into freezer bags, sealing them and strapping them to the inside of their thighs and going off to the local 'alcohol free' disco?”

(Gavin Duffy [41])

Adults, on the other hand, appear mainly in the role of influencers, for example, “*Peers and parents particularly amongst young people*, *heavily influence perceptions of ‘normal drinking behaviour”*. (Joanna Fortune, [44]) The exception again is GP Ciara Kelly, who extends the problem to the wider population:

*“But to be very clear, it isn't only young people in Ireland who have a problem with drink. Middle-aged and older people binge drink too”*.

(Ciara Kelly [35 45]).

#### (iv) The causes of excessive alcohol consumption: psychological factors, Irish culture, and peers and parents

According to the board members, there are three main causes of excessive drinking in young people: individual attitudes and motivations; Irish culture, tradition and society; and peers and parents. Psychological factors, in particular unspecified “attitudes” to drinking and “motivations” which are influenced by society, are emphasised in almost every document and interview. For example,

“As a psychologist, I have spent years looking at how attitudes and cultural influences impact our personal choices.”

(Board member Maureen Gaffney [46]).

“It is society’s psychological motivations that are driving this”

(Joanna Fortune [47]).

Other examples can be seen in S1 Appendix. This is also a major theme of the SOOCD Tweets and Facebook posts (S1 Table). Another frequently-expressed view is that ‘out of control drinking’ is due to deeply-engrained culture, e.g.:

”I don’t know whether culture informs behaviour or whether behaviour copper-fastens culture. But we all know, I think, that our historic relationship with alcohol is a deeply unhealthy one”

(Fergus Finlay [34]).

“You need to create a cultural change in society, behaviour change, and attitude change, and motivation change, and that will require more than regulation.”

[David Smith, (Diageo)*]* [37]

There are also frequent references to ‘our culture of drinking’ in the SOOCD Facebook posts, and ‘culture’ was a key theme in the public workshops (e.g. psychologist and board member Maureen Gaffney gave a talk at a workshop on “How to change culture” [46 48]).

Board members frequently highlight that it is individuals that are responsible for creating this “drinking culture”. This is done by repeating that ‘we need to take a look at ourselves’, and we need to “look in the mirror” [45] [35] [34] [49] [50]. This call for individual responsibility is also juxtaposed with the view that it is misguided to lay responsibility at the feet of the alcohol industry:

“It is the great Irish trick to seek to change everything but our own habit. So blaming the brewer, the distiller, the distributor, the publican, the legislators and the sporting organisations is a great distraction”

(Gavin Duffy [41]).

“It is very easy to call for tighter regulation of the alcohol industry, more control over prices of alcohol or more stringent ID requirements to purchase alcohol, but for every one of these valid calls I can give you real life anecdotes for how they will not stop young people drinking”

(Joanna Fortune, [44]).

“I believe there's more to it than big business manipulating us. We have a long-standing culture of excessive drinking…Peer pressure more than advertising pressure is at work amongst our teens”

(Ciara Kelly [35]).

Elsewhere David Smith (Diageo board member) rejects the influence of alcohol pricing on consumption, recommending instead a focus on individual attitudes and behaviours [32]. Ciara Kelly, however, explicitly criticises both alcohol sponsorship of sport and alcohol advertising:

“Out- of-control advertising encourages out-of-control drinking. It's the job of Government to rein in the drinks industry through legislation and regulation”

(Ciara Kelly [35]).

As in some of these quotes, peers and parents are frequently described as a key influence on young people’s consumption of alcohol [48 51]. Twenty-two of 92 Facebook posts point to individual choice and responsibility, Irish culture, tradition and society, or the influence of peers and parents (S1 Table). The ‘Call for evidence’ also refers to engaging people to think about their ‘personal drinking choices’ [36]. Elsewhere Gavin Duffy even suggests that Irish alcohol consumption might even be down to a “Celtic gene”:

“We have this view that this is a particular cultural problem in Ireland… I’d have to say Ireland and UK, but we like to convince ourselves that there is some type of Celtic gene that’s into excess- look what we did with bank borrowing for example. We do everything to excess in this country.”

Gavin Duffy [33]

### 2. Framing the solutions to ‘out of control drinking’

#### (i) “Evidence” versus “views”: evidence of effectiveness presented as just one element alongside views, conversations and experiences

When it comes to solutions to the problem of ‘out of control drinking, the need for evidence is often mentioned. However scientific evidence is frequently presented as just one element to be considered, alongside views, conversations, stories and experiences, for example:

*“Everyone’s opinions*, *experiences and attitudes towards alcohol*, *as well as the evidence put forward by professionals working in the field are included”* (Fergus Finlay [36]). Elsewhere he asks: *“Tell us your story”* [50].

Much more commonly, “views” and “opinions” are solicited without reference to evidence. For example the SOOCD Facebook page includes 30 posts (of 92) seeking public engagement with the campaign, many of which ask people to share their views e.g. “*Have you shared your thoughts on how to make a positive change to our culture of drinking*?*”* (15^th^ April 2015). The Facebook posts, tweets and other statements from board members suggest that public opinion about “what might work”, rather than scientific evidence, is central to the development of the five year action plan. Joanna Fortune’s comment above about “*real life anecdotes”* also falls into this category [44]. In this case, anecdotes are recommended as a basis to reject calls for more stringent measures on alcohol harms.

Conversely, board members generally do not recommend interventions that are consistently supported by evidence—that is, restrictions on advertising, restrictions on the availability of alcohol, and measures to increase pricing [24]. With respect to restricting advertising, board members generally do not refer to this, with the exception of Ciara Kelly who states (as noted above) *“Of course the drinks industry has enormous culpability when it comes to the fact that two thirds of Irish adults drink to excess*: *out-of-control advertising encourages out-of-control drinking*”.(Ciara Kelly [35]) In response to questioning about advertising, Diageo’s David Smith at one point replies that “*You’ve got to be careful that the legislation doesn’t end up the wrong thing*, *but the broad thrust of*, *should we not advertise to people underage*, *we completely agree*…”.[32] However it has been shown elsewhere that measures to restrict advertising to children are often ineffective, as children are widely exposed to advertising in sports, on billboards and other adverts, including in television programmes [52].

In the case of pricing, and specifically Minimum Unit Pricing, most board members do not mention it, with the exceptions of Charlie O’ Connor, who states that he has no problem with Minimum Unit Pricing, [40] and Joanna Fortune, who as noted above rebuts the suggestion that pricing is effective (Joanna Fortune, [44]). While David Smith does refer to pricing, stating “*Diageo is very*, *em*, *supportive of all of the objectives of the alcohol bill*, *and I personally believe*, *and we believe*, *that there should be regulation around pricing*…”, it appears that he is actually referring to below-cost selling, while still being in favour of a focus on psychological influences:

”*Something that …would be allowed to stop people selling alcohol…below cost*. *That would make a lot of sense…the interesting thing is*, *its considerably more expensive than they pay for a can of beer in Germany*, *or in France*, *or in Italy*, *or in Spain so it’s another good example that this goes much deeper than any individual area*, *we’ve got to get into attitudes and behaviours*”. [32]

Below-cost selling is assumed by many to be much less effective than minimum unit pricing for alcohol, as the former affects so little of the market [53]. It has been estimated that in England the current ban on below-cost selling is 40–50 times less effective than minimum unit pricing would have been [54].

#### (ii) The emphasis on the need for educational interventions

While effective interventions are generally ignored or rejected by most Board members, they place a strong emphasis on educational interventions which, as discussed earlier, are known to be ineffective. They also frequently justify the SOOCD campaign by stating that everything tried previously in Ireland has not worked. In these examples, board members specifically solicit information from the public on educational and information-based approaches:

“We want to hear your views on what you think is the best way to tackle out-of-control drinking. …What type of education, information, public awareness will have the most impact”

(Fergus Finlay[50]).

“Mass media, awareness raising, prevention and education, and initiatives that challenge certain behaviours as normal or acceptable are the most successful”

(Board member, child/family psychotherapist Joanna Fortune [44]).

The Call for Evidence, Facebook page and tweets also solicited public views on educational interventions (S1 Table) [51].

#### (iii) The legitimacy of the SOOCD campaign, and the “independent”, board

The SOOCD campaign itself is the main solution proposed by board members. However the campaign’s legitimacy was frequently challenged in media interviews, in which the interviewers often pointed to Diageo’s role. Perhaps for this reason the issue of independence is frequently emphasised in board members’ statements [48 55], including in a tweet from Fergus Finlay: “*Funded by Diageo*, *nobody tells us what to think*” [56]. Elsewhere he says: *“When a group of us came together*, *Diageo gave us a written memorandum that underpins our independence from them”* (Fergus Finlay [34]). However the Memorandum is mainly a statement of the campaign aims, and does not state how independence is to be assured [55]. The unpaid, voluntary nature of the board is also highlighted by board members themselves as evidence of their independence, and Diageo’s involvement is presented by several Board members as a form of altruism. For Rob Hartnett (quoting Theodore Roosevelt), and Gavin Duffy, the board and Diageo are engaged in an act of great daring:

““It is not the critic who counts…the credit belongs to the man who is actually in the arena…if he fails, at least fails while daring greatly.””

(Rob Hartnett/Sport for Business [38]).

“I congratulate Diageo on its bravery to back us and its genuine commitment, too, to initiating a national debate”

(Gavin Duffy [41]).

Guinness and its history in Ireland is also used to legitimise Diageo’s involvement [41 57]. For example SOOCD board Chairman Fergus Finlay wrote in a newspaper article about the campaign:

“As for Diageo, a couple of weeks ago it was a company that most of us in Ireland were pretty proud of. We know it as Arthur Guinness. It’s a company with a long and proud tradition. It runs the most famous brewery in the world–and it’s a Dublin institution.”

(Fergus Finlay [49]).

David Smith also emphasises Diageo’s commitment to the campaign aims by stating that Diageo has been in Ireland “over 250 years” [32], although Diageo, a British-based multinational, was formed in 1997. Although Guinness is mentioned by board members, Diageo’s other alcohol products (such as Smirnoff vodka) are not referred to, although spirits are the most the most common type of alcohol consumed by young Irish women [18].

It has been suggested that one of the unspoken aims of the campaign is to undermine the proposed Public Health (Alcohol Bill) Alcohol in Ireland[20]. Some board members state that they support the “framework” of the bill, or its ‘thrust’, or its general objectives (e.g. Gavin Duffy [58]), but either do not comment on, or do not support specific elements of the bill, in particular Minimum Unit Pricing (MUP). Although Fergus Finlay stated that the campaign totally supported the Bill[57], in April 2015 the SOOCD campaign failed to back MUP [59].

Several board members resigned following extensive criticism of the campaign. Dr. Ciara Kelly resigned first on 5th March 2015 citing time constraints, but stating that “*She remains a strong supporter of the campaign and encourages others to get involved at* www.rolemodels.ie *and help shape Ireland’s plan to stop out-of-control drinking*”. [60] Psychologist Krystian Fikert of the MyMind mental health centre resigned on 15^th^ March, citing resource constraints.[61] Paul Gilligan, CEO of St Patrick’s Mental Health Services, and the Diageo member, David Smith, resigned in late March. The latter stated that he had done so “*To ensure that the board members can work to the best of their abilities and carry out the objectives of the campaign without further pressure and distraction*”. [62]

It is possible that other board members also resigned either then or since without giving similar public statements, but this is unclear.

## Discussion

Our analysis shows that the SOOCD campaign has a strong focus on visible antisocial behaviour (ASB) related to alcohol, particularly among young people. By contrast, the health effects of alcohol consumption are almost entirely absent from discussion and “moderate drinking” is presented as a behavioural issue, rather than a health issue. The board members focus strongly on individual responsibility, while emphasising unspecified psychological influences, peers, parents, and Irish culture as the main influences on consumption, as opposed to industry activities such as marketing and advertising. In some cases the responsibility of industry is explicitly excluded as an influence by board members. Public opinion and views are prioritised as a source of evidence, instead of scientific evidence, and board members frequently recommend informational and educational interventions, stating inaccurately that they are known to be effective.

### The emphasis on ‘drinking behaviour’ rather than health

The construction “drinking behaviours” (or “negative drinking behaviours”) is prominent in the interviews and newspaper articles written by board members. One potential consequence of using this phrase is that it does not require asking people to drink less, because the phrase implies that it is the “negative behaviour” associated with drinking (for example, antisocial behaviour) that is the problem, not the amount consumed. Some board members do explicitly reject a focus on the amount drunk, and suggest that people should make up their own mind how much to drink. Nearly all board members also place greatest emphasis on the visible behavioural consequences of alcohol consumption, and not on the effects on health. This very specific framing of the problem as a behavioural issue allows people to drink as much as they want, guided only by self-defined limits, as long as the negative effects of their consumption are not publicly visible. It is also notable that while “culture” is repeatedly cited as an important cause of ‘out of control drinking’, and ‘negative drinking behaviour’, the role of the alcohol industry in creating and shaping that culture is not acknowledged by most board members.

The framing adopted by the SOOCD campaign is very supportive of the needs of the alcohol industry, but not of public health. It is also consistent with a wider alcohol industry strategy of focussing on visible anti-social behaviour in young people. For example, in a 2014 speech the CEO of Diageo, Ivan Menezes, defined “harmful drinking” narrowly in terms of antisocial behaviour and underage drinking [63]. The commitments outlined by the global beer, wine and spirits producers in 2015 also include a focus on underage drinking, and to “*develop*, *promote and disseminate educational materials and programmes designed to prevent and reduce underage purchase and consumption*, *which either address young people themselves or those known to have a strong influence over their behaviour*” [64]

One reason for the alcohol industry focus on underage drinking and antisocial behaviour may be that publicly visible antisocial behaviour poses a significant PR challenge, because it attracts attention in the media (as some board members have pointed out), and it negatively affects the wider public perception of the industry. At a time when new regulatory approaches to reducing alcohol harms in Ireland are being considered, it is possible that the aim of this campaign—consistent with existing evidence on industry strategies [63]–is to confuse the agenda, to reframe the problem away from the industry, and to encourage ineffective solutions in preference to more effective population-based regulatory measures. Indeed the manner in which the SOOCD campaign materials appear to encourage the public towards suggesting and discussing ineffective educational and information interventions supports this interpretation.

### Parallels with other alcohol industry activities

The tactics and framing employed by this campaign have clear parallels with well-documented alcohol industry activities internationally [14]. These activities have been shown to include: indirect lobbying in order to oppose public health measures by creating front groups, and by forming alliances with civil society organisations and consumers; the promotion of non-regulatory initiatives; a focus on individual responsibility, and the (mis)behaviour of a small minority; the omission of “health” from discussions; and misrepresentation of the evidence base [14]. Our analysis identified all of these in the SOOCD campaign.

Casswell (2013) has also noted that the industry in other countries co-hosts public meetings, including policy development workshops, in order to establish credibility as ‘part of the solution’, while focussing attention on ineffective educational approaches [12]. Public workshops are a key part of the SOOCD campaign, and the data from such workshops may also be of commercial and strategic value to industry, above and beyond their relevance to the SOOCD campaign. McCambridge (2013) has also reported that industry emphasises public opinion, while producing distorted views of the scientific evidence [5]. Both these tactics can be seen in the SOOCD campaign, where the boundaries of what constitutes scientific evidence are blurred to include views, opinions, and anecdotes.

The framing of the causes of the problem in terms of individual psychology, “Irish culture”, and peers and parents, may therefore be intended to undermine the measures outlined in the alcohol bill, particularly the restrictions on marketing, availability and advertising. A focus on “culture”, peers and the family, is a recognised industry frame, emphasised over many years by the industry-funded International Center for Alcohol Policies (ICAP–now IARD) [65] [66]. Industry-linked educational materials similarly focus strongly on individual responsibility and decision-making, and on the role of peers and family [67] [68] [69].

### Establishing the SOOCD campaign’s legitimacy

The legitimacy of the SOOCD campaign has been the subject of public debate in Ireland. The emphasis on public opinion, as well as some board members’ frequent references to their own children (data not presented) may be intended to establish the campaign’s legitimacy. Some SOOCD board members’ questioning of the legitimacy of critics, by associating them with prohibition or temperance, is another well-recognised alcohol industry tactic [14 69]. However non-drinkers can also be valuable as campaign supporters, as they lend apparent objectivity. In the SOOCD campaign, two board members appeared to occupy this role (one of them, Megan Tissington, declaring herself variously to be both a non-drinker, and a drinker)[70].

The repeated calls by board members for “partnership” can also be seen as a way of establishing the campaign’s legitimacy, by arguing that even WHO supports involvement of the alcohol industry [57]. However WHO does not accept the involvement of industry in policy-making,[71] a point which the SOOCD Chairman, Fergus Finlay has stated that he agrees with[57]. Despite this, the SOOCD campaign explicitly aims to involve itself in policymaking, as is clear from this statement: “*Once finalised*, *the plan will be presented to the government and community stakeholders for adoption and implementation over the coming five years”*. Favouring such partnerships with the industry over legislation is an explicit Diageo position,[72 73] [63] [73 74] and a common alcohol industry approach [14].

The legitimacy of the board is also emphasised through frequent statements about the independence of board members, and the voluntary nature of their involvement. However as noted above and in Table 1 some board members had previous or current links to Diageo, and/or to Guinness (which is owned by Diageo).

It is interesting to note that there are strong resonances, in both content and wording, between the SOOCD campaign materials, and Diageo Australia’s submission to the Government of Western Australia’s review of the Liquor Act in 2013 [75]. The Diageo Australia submission, like the SOOCD campaign, strongly emphasises attitudes and behaviour [75]. The Diageo Australia submission also draws on the findings of a Diageo-funded study by University College Dublin’s Geary Institute. Diageo provided 1.5 million Euro to the Geary Institute in 2006 to conduct the study, which emphasised individual psychology–particularly personality, attitudes, ‘mindsets and motivation’, and ‘cultural norms’. [76] [55] [77]. According to the Diageo Chief Executive, the company funded that particular study in order to discourage Irish policymakers from increasing alcohol taxes [3 78].

### Similarity to tobacco industry tactics

These tactics are not unique to the alcohol industry, many being used by tobacco companies over many decades[3]. In particular the central SOOCD narrative around “attitudes, motivations, and behaviours” and the involvement of psychologists has strong resonances with the notorious tobacco industry “sociological program,” which recruited behavioural scientists to develop a tobacco industry narrative around individual smokers’ motivations [79] [80].

### Study limitations and strengths

Our search may have missed some interviews, however the high degree of consistency between the board members’ statements (see S1 Appendix) suggests that this would not significantly affect our conclusions. As noted above we were also not able to determine the extent to which the SOOCD board members had previous business or other relationships with either Diageo, or with the alcohol industry in Ireland more generally. However it can be seen from Table 1 that at least eight of the board members have had some link with Diageo, Guinness or the alcohol industry. We did not contact board members for information about such links and it is not possible to fully ascertain all board members’ past/current business/consultancy or other relationships with Diageo or the alcohol industry, as the SOOCD website includes no information on conflicts of interest.

The key strength of the study is the timely, in-depth analysis of an ongoing campaign which we believe will be relevant to other countries. As well as the Australian connection (above), the SOOCD campaign share similarities with Diageo’s ‘Drink Right’ campaign in Jamaica, which also emphasises ‘negative drinking behaviour’ and self-defined limits on consumption.[81] We also provide further evidence of alcohol industry tactics—including the use of non-drinkers as “objective supporters”–and we draw attention to strong similarities between this campaign, and tobacco industry campaigns in how they develop arguments about smokers’ and drinkers’ psychological motivations, apparently as a means of deflecting responsibility from the industry.

## Conclusions

The SOOCD board has stated that: “The campaign is independent. It’s a people’s campaign” [82]. Our analysis of the evidence rejects this framing; not only did Diageo initiate and fund the campaign, but its content strongly reflects well-documented Diageo, and alcohol industry strategies. In short, the SOOCD campaign closely meets the needs of the alcohol industry, rather than public health in Ireland. Based on our analysis, and on previous evidence, the main effect of the five-year campaign may be to protect the reputation of Diageo in Ireland, while undermining the new public health bill.

Finally, although the framing and tactics we have identified are not unique to the alcohol industry, it is possible that they were not known to all the SOOCD board members. Our findings therefore suggest that there is a pressing need for wider awareness-raising, and a wider public debate, among the public, third sector organisations, and policymakers about such tactics. The absence of information about board members’ current and previous links to Diageo and Guinness is particularly concerning, and highlights the need for such debate to address issues around transparency, and conflicts of interest relating to relationships with the alcohol industry in Ireland.

Looking to the future, this analysis raises the need for wider debate and understanding about what the evidence actually shows in relation to effective action on alcohol harms. Board members repeat inaccurate statements about the evidence, and the final SOOCD report emphasises that the causes of alcohol harms are individuals, families and culture, while downplaying or ignoring the role of industry in shaping that drinking culture. The report also states that it is ‘evidence-based’, though the non-systematic review of the evidence which is included in the report strongly recommends ineffective information and educational interventions [26] [25]- while disputing the positive evidence on availability, pricing, and advertising. [26] [24 25] As noted in the Introduction, misrepresentation of the existing evidence is a well-documented alcohol industry strategy, and suggests that it is important to re-focus the debate on the scientific evidence—that is, what the evidence shows is effective in terms of population-level solutions.

Further analysis of future campaign materials and activities may be possible, as SOOCD is a five-year campaign (or possibly longer; campaign materials refer to “an initial five-year period”). However social media activity appears to have ceased, and at time of writing the SOOCD website is inaccessible. A more detailed analysis of the final SOOCD report would also be of value; such an analysis could analyse the evidence which it presents, and how it is framed. Future analyses could also examine the similarities between this campaign and future campaigns linked to Diageo and the industry more generally, including their framing of evidence, and their relationship to current and planned public health policies in the countries in which they are implemented. The reframing of alcohol harms in purely behavioural terms, while downplaying the health consequences, is also a priority for further research.

## Supporting Information

S1 Appendix Additional links and material from SOOCD website used in the analysis. (PDF)

S1 Table Facebook posts and Tweets from the SOOCD campaign. (PDF)
