# Supplementary material for: Diageo's 'Stop Out of Control Drinking' Campaign in Ireland: An Analysis
Source: PLoS One. 2016 Sep 16;11(9):e0160379. doi: 10.1371/journal.pone.0160379 (PMC5026331; doi:10.1371/journal.pone.0160379)
Supplement: S1 Appendix — (PDF) [file pone.0160379.s001.pdf]

S1 Appendix: Additional links, and material from SOOCD website used in the analysis

----

Key to Documents in this file

Section A: Links to articles from newspapers.

Section B: Links to shorter quotes and comments by board members and supporters

Section C: Relevant material from SOOCD website

Key to names:

From SOOCD media release: "Full membership of the campaign group includes:

Fergus Finlay, Chair of SOOCD board.
Gavin Duffy (Entrepreneur & TV Presenter),
Joanna Fortune (Clinical Psychotherapist),
Paul Gilligan (CEO of St Patrick's Mental Health Services), *[Resigned from SOOCD board]*
Dr Ciara Kelly (GP & Health Commentator), *[Resigned from SOOCD board]*
David Smith (Diageo), *[Resigned from SOOCD board]*
Aine Lynch (National Parents Council),
Professor Briain MacCraith (President, DCU),
Kieran Mulvey (Chief Executive of the Labour Relations Commission),
Simon Keogh (IRUPA),
Gemma Doorly (Actress, Playwright),
Anne Connolly (Director of the Irish Smart Ageing Exchange),
Rob Hartnett (Sport for Business) and
Krystian Fikert (Psychologist and Founder of MyMind)". *[Resigned from SOOCD board]*

The action plan includes a three-stage research project led by Professor Kevin Rafter, Dublin City University".

Charlie O' Connor (ex-TD) is cited elsewhere as a board member, e.g.:

http://www.independent.ie/irish-news/scrap-drinks-campaign-says-students-union-31095499.html

SECTION A: Links to newspaper articles by SOOCD board members

Document code: B2

Newspaper article by Dr. Ciara Kelly

Source: Sunday Independent

Date: 6 April 2015

Title: "Doctor's orders: Forget the controversy and concentrate on the problem"

URL:

http://www.independent.ie/life/health-wellbeing/mental-health/doctors-orders-forget-the-controversy-and-concentrate-on-the-problem-31116978.html

Reference number in main paper: 35

Project document code: B3

Newspaper article by SOOCD board Chairman, Fergus Finlay:

Source: Irish Examiner

Date: 17th Feb 2015

Title: "Time to stop papering over 'craics' and tackle corrosive alcohol abuse"

URL:

http://www.irishexaminer.com/viewpoints/columnists/fergus-finlay/time-to-stop-papering-over-craics-and-tackle-corrosive-alcohol-abuse-312921.html

Reference number in main paper: 34

Project document code: B4

Newspaper article by Fergus Finlay

Source: Irish Times

Date: Feb 23rd 2015

Title: "Everyone – even drinks companies – needs to challenge our drink culture"

URL:

http://www.irishtimes.com/opinion/everyone-even-drinks-companies-needs-to-challenge-our-drink-culture-1.2113006

Reference number in main paper: 49

Project document code: B5

Newspaper article by board member Gavin Duffy

Title: "Something must change in our booze-soaked society"

Source: Irish Independent

Date: 22nd Feb 2015

URL: http://www.independent.ie/opinion/comment/something-must-change-in-our-boozesoaked-society-31011336.html

Reference number in main paper: 41

Project document code: B6

Newspaper article by board member Joanna Fortune

Source: Galway Independent

Date: 22nd April 2015

Title: "Time to lead by positive example"

URL:
http://www.galwayindependent.com/opinion/oped/articles/2015/04/22/4064888-time-to-lead-by-positive-example/

Reference number in main paper: 44

Project document code: B7

Source: Article posted on the Sport for Business (SOOCD partner) website

Date: February 13, 2015

Title: "Alcohol and Sport. Alcohol and Society".

URL:
http://sportforbusiness.com/alcohol-and-sport-alcohol-and-society/

Project document code: B8

Newspaper article by Gemma Doorly, SOOCD board member

Title: "This much I know"

Source: Irish Examiner

Date: March 07, 2015

URL:

http://www.irishexaminer.com/lifestyle/features/this-much-i-know-gemma-doorly-actor-and-writer-316340.html

----

Project document code: B9

Newspaper article by Diageo board member, David Smith

Title: "Diageo is about more than just selling pints of Guinness: Diageo boss David Smith on why he called time on his role with the alcohol campaign"

Source: Irish Independent

Date: 29th March 2015

URL:

http://www.independent.ie/opinion/comment/diageo-is-about-more-than-just-selling-pints-of-guinness-31102593.html

----

SECTION B: Links to shorter quotes and comments by board members and supporters

Project document code: C1

Student defends 'Donegal Tuesday', telling RTE broadcaster Joe Duffy: 'February is the designated drinking month'

Source: Irish Independent

Date: 17th February 2015

URL: http://www.independent.ie/entertainment/radio/student-defends-donegal-tuesday-telling-rte-broadcaster-joe-duffy-february-is-the-designated-drinking-month-30999272.html

----

Project document code: C2

Title: "Diageo 'really believes' in tackling binge drinking "

Source: The Spirits Business

Date: 6th March 2015

URL: http://www.thespiritsbusiness.com/2015/03/diageo-really-believes-in-tackling-binge-drinking/

Project document code: C3

Statement from Fergus Finlay on Redfm radio.

Date: 25th March 2015

Original URL: http://www.redfm.ie/news/diageo-leaves-stop-control-drinking-campaign/ (note: no longer available on website)

Quote from FF:

"While he was on the board he [David Smith] was nothing but supportive and helpful. He never tried to influence us in any way, shape or form. But he recognised that a campaign was being waged around Diageo's involvement. And he wanted to kind of, you know, free us, from that., and that's what he's done."

Project document code: C4

Quote from Maureen Gaffney.

Source: Limerick Leader

Date: 19th March 2015

URL: http://www.limerickleader.ie/news/local-news/limerick-workshop-to-explore-issues-around-binge-drinking-1-6641300

Reference number in main paper: 46

Project document code: C5

Quote from Eoghan McDermott (backer of SOOCD campaign)

Source: Irish Times

Date: Feb 13th 2015

http://www.irishtimes.com/news/social-affairs/stop-out-of-control-drinking-campaign-criticised-over-diageo-link-1.2101755

Project Document code: C6

Title: “Defending Stance on Alcohol and Sport”

Source: Sport for Business website (Rob Hartnett, CEO of Sport for Business is a SOOCD board member)

Date: March 10 2015

URL: http://sportforbusiness.com/defending-our-stance-on-alcohol-and-sport/

Reference number in main paper: 38

SECTION C

Project document code: D1

Workshop materials from SOOCD website (no longer available online)

CORK PUBLIC WORKSHOP PARTICPANTS
Please use the permanent markers on the table to write down your ideas and the ideas that are being discussed on the paper tablecloth while the group discussions are happening. You will then have an opportunity to write down your own ideas on the worksheets at the end of each topic discussion. Your ideas will support the development of a five-year action plan to change the culture of drinking for the better.

Thank you for your contributions to this important discussion.

Cork Public Workshop Schedule

Time Activity
6:30- 6:40 Reception
6:40- 6:45 Welcome from Fergus Finlay
6:45- 7:20 Dr. Maureen Gaffney “How to change culture”
7:20- 7:25 Workshop explanation by Rob Hartnett
7:25- 7:40 Question 1 Discussion: Programmes and experiences
7:40- 7:45 Question 1 Write down ideas
7:45- 8:00 Question 2 Discussion: Individual/ social targets and drivers of change
8:00- 8:05 Question 2 Write down ideas
8:05- 8:20 Question 3 Discussion: Targets and drivers of change in the wider community
8:20- 8:25 Question 3 Write down ideas

8:25- 8:30 Survey & opportunity to sign-up

8:30 Thank you and close from Fergus Finlay

!

CORK PUBLIC WORKSHOP QUESTIONS

1: What campaigns, programmes or experiences have changed your attitude or behaviour, or the behaviour of someone you know?

A) What was it about the campaign, programme or experience that made it successful?

B) What other programmes or ideas would you recommend?

2: Who are the individual/ social targets and drivers of change?

A) How can we support young people to make positive choices/ change their behaviour?

B) How can we engage adults to think about or change their behaviour? What would help you?

C) How can families, peers and social network influence other people's behaviour in their networks? How can we help them?

3: Who are the targets and drivers of change in the wider community?

D) What role can organisations play (voluntary, sporting community etc.) in creating change/ supporting the above?

E) What role should government agencies and departments play in creating change/ supporting the above?

F) What can businesses do to help create change?

!

Idea Log

Please capture one idea per sheet

Ideas do not need to be fully formed

Idea

Please capture the essence of your idea

How it works

Explain briefly why it will help to stop out-of-control drinking

Have you seen/heard of this working before?

Detail any evidence you have for its effectiveness

Your details

(leave blank if you'd like to submit anonymously)

Name:………………………………………………………………………………

Email address:…………………………………………………………………….

Are you happy for us to follow up with you on your idea?

(please cross out one)

Yes / No

Campaign to Stop Out-of-Control Drinking Workshop

Survey

Name (optional):

Organisation (optional):

Email (optional):

We appreciate your feedback on the workshop and campaign. If you would like to receive updates about news and upcoming events please provide your name and email address. You can also submit comments after the event at workshops@rolemodels.ie Thank you.

Q 1: Why did you attend this workshop?

Q 2: How interesting did you find this workshop?

Very interesting

Interesting
Somewhat interesting
Not interesting

Q 3: Would you recommend this event to your friends/ family/ colleagues?
Yes
Maybe
No

Q 4: Would you attend another event held by the campaign to Stop Out- of-Control Drinking?
Yes
Maybe
No
!
Q5: Would you like to be on our mailing list? Please provide your name and email below:

Q6: Have you/ will you sign-up to support the campaign at www.rolemodels.ie
Yes
Maybe
No

Q 7: Do you have any feedback or recommendations on the workshop and/or suggestions for the campaign?

Q 8: Are there any ways you think the campaign could include more peoples' opinions or evidence?

Q 9: Any other comments?
First Name (Print)
Last Name (Print)
City
Email
Phone
Signature

The campaign to Stop Out-of-Control is bringing people together to make a positive change to our drinking culture. By signing up you are joining a growing movement, one that provides a unique opportunity to break the tolerance of alcohol misuse in Ireland, which has allowed excessive drinking to become an accepted part of Irish culture.

Sign up to show your support for a campaign that wants to make a difference. Joining is just one of the ways you can make your voice heard. Visit rolemodels.ie to find out about events near you, how to get involved and campaign competitions. Be part of the solution, join the campaign to Stop Out-of-Control Drinking now!

Please complete all the details below. Return completed form to **info@rolemodels.ie** or post it to **Stop Out-of-Control Drinking, 6-9 Trinity St, Dublin 2.**

Stop Out-of-Control Drinking | 6-9 Trinity St | Dublin 2   01 617780   info@rolemodels.ie

@Irishrolemodels
#irishrolemodels

I am 18yrs+ and opt in with consent for future campaign updates

I am interested in the following:

Providing a vox pop or photo today [If so, consent form required]

Sharing views at a public meeting in Dublin on the evening of March 5th [If so, give invite]

Two final questions:
In the last 6 months have you done something you regret as a result of your drinking?
In the last 6 months has your drinking had a negative impact on someone around you?
Your name, address, email and any personal information will not be shared with others, including other members of our campaign, other than for the purpose of listing you as a supporter. You can opt out at any time via the link provided in all communications.

The campaign to Stop Out-of-Control Drinking
The campaign to Stop Out-of-Control Drinking is an independent initiative that seeks to change our culture of drinking for the better. This campaign has brought together public figures from education, public policy, mental health, children's services and sport.

In Ireland we are all too aware of the damage that out-of-control drinking does – economically, socially, and in terms of our country's reputation. Our aim is to establish an honest and strong nationwide campaign to break the cycle of the culture of excessive drinking passing from generation to generation, and to change behaviour in a sustainable way.

Part of the reason we came together is a simple one. Despite the best efforts and total commitment of organisations, individuals, and experts and despite the obvious and transparent damage in families and communities, none of us have cracked the challenge of cultural change. We need to ask ourselves challenging questions. Why do we have this relationship with alcohol? Why do we need to drink this way? What motivates us to do this? What can we do to change this culture that generations of us have accepted? How can we implement that change?

I'm delighted to welcome you to our meeting today. We want to hear what you have to say.

We need your viewpoint and your perspectives on solving this challenge. We want to learn what works and based on the feedback we receive we will create a five-year plan, which we can build a campaign around, and one that will be evidence-based and have been informed by experts and the public.

There are also ways you can stay involved in the campaign after today's meeting including starting a conversation with us on facebook www.facebook.com/irishrolemodels) and Twitter @irishrolemodels. You can also give us more of your thoughts or make a formal submission to the action plan at www.rolemodels.ie/haveyoursay and if you haven't already, sign-up to show your support for change at www.rolemodels.ie.

Thank you very much for your contributions today. We look forward to continuing to work with you.

Warm regards,

Fergus Finlay
Chair of the campaign to Stop Out-of-Control Drinking!

Project document code: D2

Memorandum of Understanding from SOOCD website (no longer available online)

Date: 12/2/15

1. Stop Out-of-Control Drinking ('the campaign') is an independent group that wants to make a positive and lasting change to the misuse of alcohol and its consequences in Ireland, for the individual and society.

2. We recognise the serious consequences of alcohol misuse in Ireland. The links between alcohol misuse and ill health are well established, as are the consequences in terms of anti-social behaviour, dangerous driving, and dysfunctions within families. There is an overwhelming need to break this cycle.

3. We intend to lead a campaign that will be based on evidence and strong and effective messages that will influence networks and groups in society. We will challenge motivations and perception of excessive drinking as a cultural norm, asking the public to become part of a solution that focuses on preventative measures, bringing about a change in drinking behaviour.

4. The campaign is made up of a board of founder members acting in a voluntary capacity, led by a chair and supported by a secretary.

5. The campaign will work with groups and individuals from across Irish society to find what preventative approaches can help achieve this goal.

6. This work will go into an evidence-based action plan that will lay out key steps to address this issue, for both the individual and society.

7. The development of the action plan will be led by an external consultant, with the board members taking an active role in that process and will have final approval.

8. The chair acts as the spokesperson for the campaign board. The chair may also nominate other members to act as spokespeople in an official capacity.

9. New members of the board can be appointed at the discretion of the board and members may resign from the board by notifying the secretary. Membership of the campaign is subject to acceptance of this Memorandum of Understanding and membership can be rescinded at the chair's discretion.

10. The campaign and its board will not be a legal entity. Its rules, this MoU and its mission statement will be placed in the public domain.

11. Support for the campaign's activities is being provided by Diageo Ireland.

Embargo: 00:01 12 February 2015

Project document code: D3

Website advert for SOOCD public workshops

Sport for Business is supporting a major national campaign to Stop Out of Control Drinking.

The first public workshop looking for ideas and thoughts from the broad base of the general public on how we can face up to our relationship with drink takes place in Dublin on March 5th.

Maureen Gaffney, best-selling author and clinical psychologist will lead a discussion on the influence that cultural perceptions and attitudes can have on our relationship with alcohol. It's then over to you to let us know what you'd change to create a better drinking culture. A light dinner will be provided.

Come and join us and have your say on the debate.

Project document code: D4

Anne Connolly (SOOCD board member), quote from SOOCD Website

Anne Connolly, board member and Director of the Irish Smart Ageing Exchange encouraged individuals and organisations across the health, social and behavioural science sectors to make their contributions to the Action Plan.

"We need a comprehensive, evidence based, and sustainable approach to changing behaviour. We want to add to the current and past conversations and really encourage people from across sectors to share their knowledge and expertise. We need to develop a policy that will impact behaviour, as well as attitudes, to create lasting change," Ms Connolly said.

Project document code: D5

Text from SOOCD website (no longer available online)

What does drinking mean in Ireland? The stereotype of the Irish drinker is well known – but what truth is behind it, and how do you see our relationship with alcohol? We want to dig deeper and have a real conversation about drinking and its influence in Ireland. The evening will begin with a short talk from a leading social commentator about how cultural perceptions and attitudes of drinking might influence our relationship with alcohol. It's then over to you to let us know what you'd change. The discussion on the night will help build a five-year Action Plan that we're writing to change Ireland's relationship with alcohol for the better. This workshop is open to everyone, 18+. A light dinner will also be provided – RSVP with your full name and the workshop you wish to attend to workshops@rolemodels.ie to hold your place.

From the website:

The campaign to Stop Out-of-Control Drinking has today called for policy experts, academics, behavioural psychologists, health and charity professionals to put forward their evidence, research and recommendations for a five-year Action Plan.

The Action Plan will draw from secondary research, new evidence collected through an extensive public consultation and international best-practice, to deliver a strategy that addresses the motivations and influences of out-of-control drinking in Ireland.

The Chair of the campaign, Fergus Finlay, said it was important that as many people got involved in the consultation process as possible.

"We want this to be an open, inclusive and thorough process. We want to give as many people as possible the chance to contribute. It will be a truly national plan that ensures that everyone's opinions, experiences and attitudes towards alcohol, as well as the evidence put forward by professionals working in the field are

included. For change to be realised, we need to have the public on board and sharing their views on how best to approach this challenge," Mr Finlay said.

Workshops, will now be held across Dublin, Galway, Limerick and Cork during March 2015. The workshops will provide the opportunity for people to share their views, for new evidence to be collected and for organisations to highlight areas of practice that have been effective in creating sustainable behaviour change.

Anne Connolly, board member and Director of the Irish Smart Ageing Exchange encouraged individuals and organisations across the health, social and behavioural science sectors to make their contributions to the Action Plan.

"We need a comprehensive, evidence based, and sustainable approach to changing behaviour. We want to add to the current and past conversations and really encourage people from across sectors to share their knowledge and expertise. We need to develop a policy that will impact behaviour, as well as attitudes, to create lasting change," Ms Connolly said.

Project document code: D6

Text from SOOCD website (no longer available online)

D6 SOOCD Events

Our country-wide consultation will create a five-year action plan to change our culture of drinking for the better.

For more information or to hold your place email workshops@rolemodels.ie

Please see a list of event dates and details for Dublin, Galway, Limerick and Cork below.

Public Workshop: Maureen Gaffney, best-selling author and clinical psychologist will lead a discussion on the influence that cultural perceptions and attitudes can have on our relationship with alcohol. It's then over to you to let us know what you'd change to create a better drinking culture. A light dinner will be provided.

Policy Workshop: This workshop will collect research, evidence and experience from professionals and organisations. It will be hosted by Fergus Finlay, CEO of Barnardos and Chair of the campaign to Stop Out-of-Control Drinking. Refreshments will be provided.

Don't miss your chance to impact the debate.

UPCOMING

Cork Policy Workshop

Date: 26th March 2015

Time: 2:30pm – 5:00pm

Venue: Morgan & Plunkett Suite, Imperial Hotel, South Mall, Cork

RSVP by: 24th March 2015

Cork Public Workshop

Date: 26th March 2015

Time: 6:30pm – 8:30pm

Venue: Corcaigh Suite, Cork County Hall, Carrigrohane Rd, Cork

RSVP by: 25th March 2015

PAST

Dublin Policy Workshop: 4th March 2015

Dublin Public Workshop: 5th March 2015

Galway Policy Workshop: 12th March 2015

Galway Public Workshop: 12th March 2015

Limerick Policy Workshop: 19th March 2015

Limerick Public Workshop: 19th March 2015

Project document code: D7

Text from SOOCD website (no longer available online)

Campaign Aims

We're saying enough is enough and we need to stop out-of-control drinking being considered an acceptable part of life. We want to break the cycle of passing a cultural acceptance of excessive drinking from one generation to the next.

We cannot address this problem by working alone. We must work together as individuals, organisations, and society to find a lasting solution to out-of-control drinking.

Our three-part campaign is built on sound evidence and thorough research, and aims to provide a permanent change in our drinking behaviour.

Raising Awareness

Our campaign will raise awareness of the consequences of drinking to excess, the damage it can do to the individual and the impact it can have on others. The campaign will challenge society to think about what is and isn't socially acceptable. It's unbelievable to us now, but drink driving was socially acceptable not so long ago, with the right motivations, positive cultural change can happen.

See the adverts here.

Engagement

Evidence suggests that to make the lasting difference needed, we must alter the attitudes and motivations that lie behind our drinking behaviour – our campaign seeks to understand those motivating factors.

What is really clear from all the evidence is that each cultural attitude towards alcohol will require a different response if we are to achieve long-term behaviour change. What works in one country may not work in another, we need a tailored Irish response.

In the next stage of our campaign we will travel across the country to find out what will really make a difference – by discussing the issues with local people and groups of acknowledged experts in alcohol policy and behaviour change.

Building the long-term solution

Following our consultation with international and Irish experts, and considering the views of the Irish public on what factors would influence their behaviour, we will then draw up a five-year plan to change our drinking culture for good. It will set out a concrete set of actions that we as individuals, organisations, and society can take, to help eliminate out-of-control drinking.

Have your say now and help shape the plan here.

Project document code: D8

Fergus Finlay, text on SOOCD website (no longer available online)

"Today we launch our campaign"

I'm delighted to welcome you to the campaign to Stop Out-of-Control Drinking. Our campaign is aimed at changing Ireland's culture of drinking for the better. We want to change attitudes and behaviours forever. We want you involved. This is your chance to join, shape and lead a campaign aimed at one of our most pressing social issues.

Throughout my professional life, I've seen the damage that alcohol abuse can do. It damages families and it damages children. It causes economic damage and of course it helps to define our reputation in all sorts of damaging ways.

So. Out-of-control drinking is a serious issue, with enormous consequences. To stop it we need to work together as a nation. We need to make a collective decision that enough is enough, that we want things to be better, that instead of being part of the problem we can be part of the solution. Working together we can make it better for ourselves, for our families, for our society both today and for the generations that will follow us.

This will be an honest and strong campaign that will hold a mirror up to something that is unacceptable to Irish society. We are starting a process that will take a number of years and we're determined that it will play a serious part in achieving that goal together, by creating a well-considered plan for getting to grips with something that is deeply ingrained in our culture.

We want to create the kind of lasting impact on attitudes to excessive drinking that has been achieved with drink driving.

At the same time as asking people to consider their own drinking habits, this campaign will develop a long-term plan to bring about that cultural and behavioural change. We will be seeking out best practice, both at home and abroad, to find out what will work best for Ireland. We will be hearing from experts and professionals what changes we need to make to achieve our aims. Out-of-control drinking isn't just an issue for the government, of course, it's a problem that affects all of us, and a problem for society needs a solution that involves everyone. So our campaign will be throwing this open to the public.

We want to hear your views on what you think is the best way to tackle out-of-control drinking. Tell us your story. Help us to understand the impact out-of-control drinking has had on you. Tell us what's made you consider your own drinking? Who should we be talking to? How do we stop this attitude towards alcohol passing from generation to generation. What type of education, information, public awareness will have the most impact. To have the kind of impact we want and to make that positive change, we need your help, and there are so many ways to contribute for a better future for all of us. Show your support for a better culture of drinking by joining the campaign to Stop Out-of-Control Drinking today. Get involved and have your say.

- *Fergus Finlay, Chair, Stop Out-of-Control Drinking*

Project document code: D9

Text from SOOCD website (no longer available).

The campaign to Stop Out-of-Control is bringing people together to make a positive change to our drinking culture. By signing up you are joining a growing movement, one that provides a unique opportunity to break the tolerance of alcohol misuse in Ireland, which has allowed excessive drinking to become an accepted part of Irish culture.

Sign up to show your support for a campaign that wants to make a difference. Joining is just one of the ways you can make your voice heard. Visit rolemodels.ie to find out about events near you, how to get involved and campaign competitions. Be part of the solution, join the campaign to Stop Out-of-Control Drinking now!

Please complete all the details below. Return completed form to info@rolemodels.ie or post it to Stop Out-of-Control Drinking, 6-9 Trinity St, Dublin 2.

Project document code: D10

Media release

New Campaign Launched to Stop Out-of-Control Drinking
-Children's groups, parents, medics and the education community join celebrities and sportspeople to stop out-of-control drinking.

-Board members include Fergus Finlay (Chair) , Dr Ciara Kelly and Gavin Duffy. Campaign supporters include Kian Egan, Eoghan McDermott & Una Foden.

-Irish public urged to join campaign and help create an unprecedented national plan to improve Irish drinking culture.

12 February 2015: A new independent campaign to change Ireland's drinking culture and behavior was launched today. Chaired by Fergus Finlay, the campaign to *Stop Out-of-Control Drinking* challenges the public to get involved in a new movement aimed at tackling excessive drinking culture in Irish society, and changing behavior in a sustainable way.
The campaign is funded by Diageo but led by an independent campaign board of figures as diverse as Dr Ciara Kelly (GP & Health Commentator), Paul Gilligan (CEO of St Patrick's Mental Health Services), Gavin Duffy

(Entrepreneur & TV Presenter), David Smith (Diageo), Áine Lynch (National Parents Council) and Kieran Mulvey (Chief Executive of the Labour Relations Commission).

It is also has the backing of celebrities such as Kian Egan, Una Foden, Ardal O'Hanlon and Eoghan McDermott. The campaign aims to achieve the same shift in attitudes towards excessive drinking culture as there has been towards drink driving within the last 20 years. The campaign will call on the public, organisations and experts to join online and at public meetings across the country, to generate an unprecedented national response to this issue. That response will form the heart of a "people's campaign" and will shape the creation of a society-led action plan to make out-of-control drinking an unacceptable part of Irish culture by 2021. The plan will be published in Summer 2015.

Fergus Finlay, the campaign's chair said:
"Out-of-control drinking is a serious issue in our society, with enormous consequences. To stop it we need to work together as a nation. We need to make a collective decision that enough is enough, that we want things to be better, that instead of being part of the problem we can be part of the solution. Working together we can make it better for ourselves, for our families, for our society both today and for the generations that will follow us. This will be an honest and strong campaign that will hold a mirror up to something that is unacceptable to Irish society. We are starting a process that will take a number of years and we're determined that it will play a serious part in achieving that goal together, by creating a well-considered plan for getting to grips with something that is deeply ingrained in our culture."

Dr Ciara Kelly, GP and Health Commentator, said:
"Every day I see the impact that excessive drinking has on the wellbeing of the nation. But there is a deeper cultural issue here that needs to be addressed. If we can find a way to change our attitude towards drinking, we can prevent the health impacts before they happen. That is why this campaign is so important."

David Smith, Director of Diageo Ireland and campaign board member said:
"This is a campaign that we at Diageo really believe in. We have always sought to work in partnership with others on the issue of alcohol misuse. Real cultural change can only happen with collaboration."

Una Foden, musician and coach on The Voice of Ireland, said:
"With a young family, I have a keen eye for what the future holds for my children. It would be great to make a positive difference to Ireland's future health by getting a grip on our drinking culture today. This campaign presents us with that chance. Join the campaign and let's take it together."

ENDS
About the campaign to Stop Out -of-Control Dr inking:
For more information contact Conor Dempsey, M: 086-247 9892, E: media@rolemodels.ie
Notes to Editors:

More information on the campaign and the action plan can be found at www.rolemodels.ie

Full membership of the campaign group includes: Gavin Duffy (Entrepreneur & TV Presenter), Joanna Fortune (Clinical Psychotherapist), Paul Gilligan (CEO of St Patrick's Mental Health Services), Dr Ciara Kelly (GP & Health Commentator), David Smith (Diageo), Aine Lynch (National Parents Council), Professor Briain MacCraith (President, DCU), Kieran Mulvey (Chief Executive of the Labour Relations Commission), Simon Keogh (IRUPA), Gemma Doorly (Actress, Playwright), Anne Connolly (Director of the Irish Smart Ageing Exchange), Rob Hartnett (Sport for Business) and Krystian Fikert (Psychologist and Founder of MyMind).

The action plan includes a three-stage research project led by Professor Kevin Rafter, Dublin City University.
The campaign will hold eight workshops/public meetings in Cork, Dublin, Galway and Limerick during March.
More information and dates available on request or via the website.
The campaign will include an online, broadcast and outdoor awareness raising campaign – visuals

Eoghan McDermott, radio DJ and presenter of The Voice of Ireland, said:

“We have a strange attitude towards drinking. We think it is normal to lose control of ourselves. Even stranger, we tolerate people who lose control of themselves when drinking, no matter what the consequences. We don’t need to change altogether but we do need to cop on. This campaign might help us do that.”

Ardal O’Hanlon, actor and comedian, said:
“When I'm abroad, I hate the way people immediately identify the Irish with drink, and out-of-control drinking at that. It's tedious and embarrassing and far from the full picture. (We're quite good at tunneling and poetry as well!) One way we could improve our image would be to curb our excesses and this campaign might well be a step in the right direction."

Kian Egan, musician and coach on The Voice of Ireland, said:
“I have been lucky enough to have travelled the world in my career and I can see that we are out of line in our approach to drinking. I would prefer my children to feel less pressure and more in control about drinking alcohol, so I am getting involved for them as well as for myself.”
Other supporters include: Dr Eamonn Brazil and Shane Byrne among others.

Project document code: D11

The Campaign to Stop Out-of-Control

Drinking Action Plan: Call for Evidence

Launched: 12th February 2015

Contact: actionplan@rolemodels.ie

1. Introduction

While consumption of alcohol continues to decrease in Ireland, consequences arising from alcohol misuse remain a significant concern. The campaign to Stop Out-of-Control Drinking was launched on February 12th 2015 to bring together a wide range of people, opinions and expertise from across Ireland to reduce the prevalence of excessive drinking behaviour and change Ireland’s relationship with alcohol for the better.

Over 5 months, the campaign to Stop Out-of-Control Drinking will work with the Irish community to develop and deliver a five-year, community-wide action plan that can begin to reduce excessive behaviour. Once finalised, the plan will be presented to the government and community stakeholders for adoption and implementation over the coming five years.

The call for evidence invites everyone with an interest in changing the culture of alcohol consumption in Ireland to share their views, knowledge, evidence and expertise.

We are interested in hearing about specific strategies, programmes and methods of delivery that have been effective in changing behaviour related to alcohol consumption, including preventative tactics and programmes that may also be applied to broader sections of society. We are also interested to hear about evidence-informed but currently untested methods for change: your recommendations on how best to a) engage people who view excessive alcohol consumption as ‘the norm’ to think about their personal drinking choices b) how to engage people to consider the impact their drinking behaviour has on others, and c) suggestions on how to ensure consistency across Ireland in the delivery of education based and awareness raising initiatives.

The intention of this action plan is not to replicate the work of other organisations in this field, but to collate the breadth of information and experiences drawn from the Irish population and deliver a strategy that builds upon previous conversations; notably, there has been no cross-sector, community-wide strategy that offers a sustainable, long-term and comprehensive solution that addresses the social and cultural challenges faced in Ireland. Therefore, the plan will draw from a range of primary and secondary research, international best practice and untapped domestic evidence, then collate and present this to deliver a comprehensive, cross-

sector and multi-track strategy targeting the causes and motivations of excessive drinking across society as a whole. Most importantly, we are seeking input from the wider Irish population –people with professional

expertise, but also anyone with personal experience of the drinking culture, in order to shape a policy that will work for all the people of Ireland.

2. The Action Plan

2.1 Overview

The purpose of the action plan is to:

Provide a comprehensive, cross-sector, population-wide and long-term approach to changing behaviour related to heavy episodic alcohol consumption, starting with an initial five-year period.

Identify and recommend evidence-based and evidence-informed strategies to reduce the prevalence of excessive and heavy episodic drinking behaviour and to engage broad and often overlooked, sections of society.

Identify and recommend strategies and programs that may be adopted, developed and/or *extended* by governments or community organisations.

Develop an effective community led, policy response that focuses on influencing the social and cultural motivations behind alcohol misuse.

As part of the evidence gathering stage the campaign to Stop Out-of-Control Drinking group will:

Undertake an extensive research phase and literature review.

Collate and analyse formal submissions received.

Collate, analyse and weight informal submissions received.

Undertake a consultative workshop phase with workshops in Dublin,

Galway, Limerick and Cork consisting of a) a professional workshop b) a public workshop to collect feedback and evidence, and generate ideas.

Refine the report by conducting a secondary submission stage through public responses to four specific questions to be defined at a later date.

What we are looking for in the submissions:

Evidence and case studies of successful strategies and programmes that have reduced or prevented excessive alcohol consumption.

Evidence-based and evidence-informed recommendations for behavioural, social and cultural change including methods to engage individuals across all age groups who:

o drink heavily on single-occasions

o consider excessive consumption of alcohol ‘normal’ drinking behaviour

Integrated strategies that work across a number of mediums

Education, prevention and awareness raising strategies and recommendations including efficacy building programmes and population-wide advertising campaigns, workshops and programmes.

Strategies from other sectors/ fields that can be applied to changing social and cultural behaviour related to alcohol.

Strategies to enable consistency of delivery and sustainability of programs to ensure long-term success as well as short-term.

We encourage you to think beyond current parameters and limitations when providing recommendations for future strategies.

2.3 How to respond

Please send your formal submissions electronically to actionplan@rolemodels.ie. Please do not feel that you need to complete all the questions; responses to any of the questions or submissions that address some of the questions outlined below are also encouraged.

For any queries please email actionplan@rolemodels.ie. We will respond to your query as soon as possible.

3. Each submission should include

Name of organisation, contact person and title.

Details about your organisation, it's function and experience dealing with behaviour change, alcohol policy, and/or delivering interventions or programmes that influence individual decisions or cultural change.

What is your interest in contributing to the action plan?

Information on current/ past programmes, campaigns, services and research, outlining, where possible:

o The purpose, key aims, target groups and KPIs.

o Length of programme.

o Results and key lessons.

o Any challenges in impacting attitudes/ behaviours.

o Recommendations as to whether, why and how the programme could be rolled out:

o To a different target group

o Wider audience

o In Ireland, regionally or nationally.

o Please provide any evidence, evaluations, key statistics or case studies you think would be relevant.

o Where possible, please provide an online link to any programme referred to. Please give the title of any reports or studies undertaken on this specific programme.

4. Questions, evidence and recommendations

4.1 Motivations and influences

In your professional experience:

o What are the motivations for heavy episodic drinking in the people that you work with (please specify age groups, nature of interactions etc.)?

o What are the influences that impact these individuals’ decisions to a) drink moderately, heavily or choose not to drink and b) their attitudes and perceptions of drinking and/or excessive drinking?

o Why?

Aside from those mentioned in section 3, what programmes, strategies, services etc. are you aware of (including international programmes) that have had a positive impact on behaviour change regarding alcohol consumption or another behaviour (if another behaviour, how could it be applied to alcohol consumption?)

o What are the key components, design elements, limitations, lessons and recommendations arising out of these programmes and studies?

o Please identify, if/ where/ how they could be applied to other target groups, applied population-wide, or rolled out regionally and/or nationally.

What specific tools, programmes, campaigns, or services would you recommend to create a population-wide change in attitudes and behaviours around excessive drinking and anti-social behaviour.

Please outline your recommendations, explain why and what impact they would have and provide any relevant evidence.

4.2 Role of individuals, organisations and the government

How can organisations support individuals to make positive decisions regarding alcohol use?

What local provisions, programmes, campaigns, services, activities could engage people, including adults, and those who do not currently use government services to think about or change their drinking behaviour?

What measures could change the culture around drinking in the wider adult community?

How could people be engaged to think about their drinking behaviour who:

o do not recognise the government drinking guidelines as realistic

o do not view their drinking as a problem?

Do you think that the government has a role to play in changing the culture of drinking?

o What are the most effective roles for government to play?

o What policies are currently operating that are effective?

o What more could the government provide?

o Where are community, charity and private sector organisations better able to provide services or create change, with the right support?

o What are the responsibilities of the private sector and the drinks industry in influencing behavior change?

o Are there any other individuals/organisations that should play role in changing culture?

4.3 Partnerships and local solutions

Where are there gaps in community, organisational, local authority provision to support organisations and individuals to change attitudes, to raise awareness, to adopt safe and responsible drinking behaviour, to reduce alcohol harms, to provide alternatives to drinking and effectively deliver education and preventative measures?

How can different sectors work more effectively together to develop local and national approaches to supporting change?

What would assist organisations and individuals in delivering multitrack services, extending services to offer to the wider community, or to link up to the work of other organisations to create a more comprehensive approach to changing drinking culture in the community?

4.4 Social and cultural influences

How could attitude and perceptions that encourage the perception of excessive drinking as ‘normal’ be changed? What methods, programmes or outreach would enable this?

Who are the biggest influencers on individual drinking choices and behaviours?

o How could initiatives/ organisations/ campaigns etc. ensure that influencers are supporting positive rather than negative behaviour and individual decision-making?

o What campaigns, programmes, strategies, initiatives would enable this?

o What programmes are already running and what evidence of effectiveness exists?

What are the responsibilities of parents and peers and how can they be encouraged to think about the consequences and impacts of their behaviour?

How can schools/university-based education programmes that are offered to young people and to adults to prevent negative drinking behaviours be improved?

Consider:

o What approaches have been taken?

o Where have they been successful, where have they been less effective? Why?

o How to ensure consistency across schools, counties etc.

o How to ensure long-term impact on behaviour, once access to the education ends.

Is there a correct age at which parents and/ or educators should start involving young people in a discussion around alcohol?

4.5 Identifying evidence gaps and developing sustainable solutions

What do you see as the gaps in evidence around alcohol consumption, behavioural change and effective alcohol policies? How could these gaps be addressed?

Alternatively, what information or evidence would you like to see? What research do you think would be helpful to progress alcohol policy?

How can governments/ organisations ensure that appropriate data is developed and collected at regular intervals?

What are the key measurements and processes needed to accurately measure:

o a) alcohol related harm

o b) frequency of drinking and binge- drinking

o c) attitudes and perceptions towards alcohol

o d) changes and trends in consumption.

How could policies and strategies that ensure positive cultural and social norms around safe drinking behaviours be developed and maintained, irrespective of changes in government/ government policy?

5. Any other information or supporting evidence

Through this call for evidence, the campaign seeks to collect evidence from a wide range of sources that can help in the development of a strategy that addresses the core motivations and influences behind excessive alcohol consumption. Please provide any additional research or information that you feel should be considered. All input is welcome.

6. Publication of submissions

Most submissions will be used to inform the report, rather than the content being published. However, where case studies or data are of relevance, the information submitted to us for the purpose of the report may be used in part or in full in the final report. Unless otherwise clearly stated when making the submission, you legally agree to have your information published.

If you wish for the information to remain confidential, or attributed anonymously please make this known at the time of the submission.

The final report will be published on the campaign to Stop Out-Of-Control Drinking website.

Project document code: D12

Aine Lynch from SOOCD website

http://www.rolemodels.ie/calls-for-change-to-culture-of-out-of-control-drinking/

Chief Executive of the National Parents Council, Áine Lynch, has called for a coordinated approach to changing the culture of drinking in Ireland. Speaking at the launch of the campaign to Stop Out-Of-Control Drinking, Ms Lynch called parents across the country to give their views on how to they can better support alcohol education at home.

"The current attitude in Ireland towards out-of-control drinking is not healthy and it is time it is challenged. We know from decades of research that parents' involvement in their child's education plays a crucial role in successful outcomes for children at school. So if excessive drinking is an issue for one or both parents in a family, it can significantly impact on their ability to engage with their children's education, having the potential to have a lasting impact on their child's life." Ms Lynch said.

"This is why a campaign like this is so important. We must all do more to challenge our attitudes towards alcohol and how that impacts on those around us. We must also have a joined up approach. Education at home must be complemented elsewhere and each sector of society; government, education, industry, parents and local communities must look at their role and how they can play their part."

Aine Lynch (Board Member):

We must all do more to challenge our attitudes towards alcohol and how that impacts on those around us
Áine Lynch: http://twtrland.com/profile/Irishrolemodels

Project document code: B1

"Challenging the norm" by Megan Tissington

Source: Blog on SOOCD website (no longer available online)

Date: Undated

Reference number in main paper: 70

As a student in Ireland who doesn't drink, I get an awful lot of "ah go on, have a lush" from my drinking friends, but it doesn't really phase me. I have my reasons for not drinking – two years ago my mother passed away as a direct result of being an alcoholic, but that isn't even my main reason; the best reason I could give for not drinking is that it simply doesn't suit me. So about a year ago I made a conscious decision to give up the drink and I feel that I'm much better off for it. That's not to say that I don't have a drink the odd time, but only when I feel like it, not when others pressure me to drink, and I would never get absolutely blinded anymore, I just don't see the point.

I think the main problem is that drinking as an activity is far too acceptable in this country, and that's very clear in the attitude towards the non-drinkers like myself. If you're a non-drinker then you're considered not normal *or* weird. I have friends who, like me, know that drink doesn't really work for them but they continue to participate because that's the socially accepted thing to do. This is the attitude that we need to change, people need to start realising the influence that social pressure has on their decision to drink and begin to make decisions based not on 'what the lads are doing', but on what they themselves truly feel is right.

I don't think people truly realise the influence that their peers have on their own decisions, on the surface they probably feel like the choices they make are fully their own but I'm almost certain that if they had to drink on by themselves because nobody else was doing it, the experience would suddenly become somewhat less enjoyable. It's a difficult thing to change this mind-set, particularly when it's almost a national phenomenon, only through educating our youth can we really bring about any form of change. At the moment there is little or no form of education about alcohol and its dangers in our schools, and it's not something that the majority of parents are willing to discuss with their children, even teenagers. Educating our youth at an early age will not only lead to them having an awareness of the dangers of alcohol but allow them to make an informed decision about their own drinking habits if and when they decide to partake.

I'm not an advocate for cutting out drinking all together, it can after all be quite an enjoyable social experience, but I do believe that we need more education so that people can enjoy alcohol in moderation. This needs to be done through multiple platforms to reach people of all ages, but most importantly, it needs to be done over a larger period of time than typical campaigns in order to make sure the message reaches everybody.

This is an issue very close to my heart and it's high time we got the whole country thinking and acting on the influence alcohol is having on our nation.

– Megan Tissington
