# Supplementary material for: Diageo's 'Stop Out of Control Drinking' Campaign in Ireland: An Analysis
Source: PLoS One. 2016 Sep 16;11(9):e0160379. doi: 10.1371/journal.pone.0160379 (PMC5026331; doi:10.1371/journal.pone.0160379)
Supplement: S1 Table — (PDF) [file pone.0160379.s002.pdf]

S1 Table: Facebook and Twitter posts during Diageo's "Stop Out of Control Drinking" (SOOCD) campaign in Ireland

Theme, Subtheme	Number of Facebook posts, Tweets on each subtheme	Examples of (i) Facebook posts (ii) Tweets
**1. Framing the problem**		
Defining 'out-of-control drinking' as antisocial behaviour as opposed to health	5, 6	(i) 16th March 2015: "Have you heard our members on the radio? Tell us what out-of-control drinking means to you and how to be put a stop to it #irishrolemodels" (ii) 17th February, 2015 "Do we have a responsibility to think about how our drinking behaviour influences others? Let us know what you think "
(Re)defining moderate drinking/ binge drinking/units/guidelines	0	No Facebook posts or Tweets
Who is affected: Targeting binge drinking, underage drinking vs population harms	6, 3	(i) 20th February 2015: "Any initiative that helps to tackle excessive drinking and its effects on society and especially the young is welcome.... success or otherwise will be seen by future behaviour around drinking." – Post of quote from the Irish Times. (ii) 25th March 2015: "I joined because I don't like to see teenagers/young adults paralytic drunk. You can have as much fun without it"
Individual psychological influences & choices, Irish culture, peers and parents; Need to place less emphasis on the responsibility of industry	22, 11	(i) 23rd April 2015: "How do you think we can encourage people to think about how much they are drinking?"; 30th March 2015 "How common is peer and social pressure to drink? Share your experiences with us …" (ii) March 4th, 2015: "At the Dublin policy workshop "binge drinking is not exclusively an Irish issue" What can we learn/share with others?"
**2. Framing the solutions: Dealing with the problem/interventions/evidence**		
The need to address "attitudes, motivations, behaviours"	6, 14	(i) 6th April 2015: "What are the things that would make you change your drinking behaviour? Add your thoughts and take part in the creation of an action plan that will work for us all" (ii) February 13th, 2015: "How can we start to change attitudes & behaviours around drinking for the better?"
"Evidence" versus views, conversations and experiences.	24, 13	(i) 12th February 2015: "We're working with the people of Ireland to change our culture of drinking for the better. Join us online and at public workshops to help us find the solution." (ii) March 25th, 2015: "'We've started a national debate... It's now time to turn those conversations into actions"
Need for educational interventions (including mass media campaigns)	8, 7	(i) 21st April 2015: "Can you think of a television, online or radio advertisement or campaign that has influenced your behaviour in a positive way e.g. anti-drink driving, sunsmart, workplace safety etc.? Share them with us!" (ii) March 26th, 2015: "Have your children been educated about alcohol awareness at home or at school? Share your experiences"
The "independent", "voluntary"board;	0, 2	(ii) March 5th, 2015: "We want to develop a plan that will challenge people, industry, civil society."
What the board thinks about regulation and the new Public Health (Alcohol) Bill.	0	No Facebook posts or Tweets
